# Association of future cancer metastases with fibroblast activation protein-α: a systematic review and meta-analysis

**DOI:** 10.3389/fonc.2024.1339050

**Published:** 2024-05-01

**Authors:** Majid Janani, Amirhoushang Poorkhani, Taghi Amiriani, Ghazaleh Donyadideh, Farahnazsadat Ahmadi, Yalda Jorjanisorkhankalateh, Fereshteh Beheshti-Nia, Zahra Kalaei, Morad Roudbaraki, Mahsa Soltani, Vahid Khori, Ali Mohammad Alizadeh

**Affiliations:** ^1^ Breast Disease Research Center, Cancer Institute, Tehran University of Medical Sciences, Tehran, Iran; ^2^ Ischemic Disorders Research Center, Golestan University of Medical Sciences, Gorgan, Iran; ^3^ Metabolic Syndrome Research Center, School of Medicine, Mashhad University of Medical Sciences, Mashhad, Iran; ^4^ Department of Epidemiology and Biostatistics, School of Public Health, Isfahan University of Medical Sciences, Isfahan, Iran; ^5^ Cancer Research Center, Cancer Institute, Tehran University of Medical Sciences, Tehran, Iran; ^6^ Laboratory of Cell Physiology, Inserm U1003, University of Lille, Villeneuve d’Ascq, France

**Keywords:** fibroblast activation protein, association, meta-analysis, metastasis, cancer

## Abstract

**Introduction:**

Fibroblast activation protein-α (FAP-α) is a vital surface marker of cancer-associated fibroblasts, and its high expression is associated with a higher tumor grade and metastasis. A systematic review and a meta-analysis were performed to associate future metastasis with FAP-α expression in cancer.

**Methods:**

In our meta-analysis, relevant studies published before 20 February 2024 were systematically searched through online databases that included PubMed, Scopus, and Web of Science. The association between FAP-α expression and metastasis, including distant metastasis, lymph node metastasis, blood vessel invasion, vascular invasion, and neural invasion, was evaluated. A pooled odds ratio (OR) with 95% confidence intervals (CI) was reported as the measure of association.

**Results:**

A total of 28meta-analysis. The random-effects model for five parameters showed that a high FAP-α expression was associated with blood vessel invasion (OR: 3.04, 95% CI: 1.54–5.99, *I* ^2^ = 63%, *P* = 0.001), lymphovascular invasion (OR: 3.56, 95% CI: 2.14–5.93, *I* ^2^ = 0.00%, *P* < 0.001), lymph node metastasis (OR: 2.73, 95% CI: 1.96–3.81, *I* ^2^ = 65%, *P* < 0.001), and distant metastasis (OR: 2.59; 95% CI: 1.16–5.79, *I* ^2^ = 81%, *P* < 0.001). However, our analysis showed no statistically significant association between high FAP-α expression and neural invasion (OR: 1.57, 95% CI: 0.84–2.93, *I* ^2^ = 38%, *P* = 0.161).

**Conclusions:**

This meta-analysis indicated that cancer cells with a high FAP-α expression have a higher risk of metastasis than those with a low FAP-α expression. These findings support the potential importance of FAP-α as a biomarker for cancer metastasis prediction.

## 1 Introduction

Metastasis is the process by which cancer cells escape from the primary tumor location and colonize distant tissues. It is responsible for more than 90% of cancer deaths, making it a worthwhile goal in cancer therapy (1). The mechanisms leading to the multistep processes, from local invasion at the primary site to metastatic expansion at the secondary site, remain obscure. It has become apparent that the tumor microenvironment (TME) can play a dynamic role in modulating the motility and hostility of cancer cells in metastatic tissues (2). In this respect, TME can involve the extracellular matrix and basement membrane, endothelial cells, cancer-associated fibroblasts (CAFs), neuroendocrine cells, and signaling pathway molecules that regulate tumor development and metastasis (2). CAFs are the most common tumor stromal cells in TEM homeostasis. Studies have reported different origins or predecessors of CAFs, including resident tissue fibroblasts, bone marrow-derived mesenchymal stem cells, hematopoietic stem cells, and endothelial cells. It is possible to distinguish different subtypes of CAFs based on certain stromal markers, such as fibroblast activation protein-α (FAP-α), integrin β1, and α-smooth muscle actin (3). Among these, FAP-α, or seprase, is a vital surface marker belonging to prolyl-specific serine proteases (4). It is not detectable in healthy adult tissues outside of tissue remodeling or wound healing areas. FAP-α is highly expressed on the surface of CAFs surrounding epithelial cancer cells, including breast, colon, ovarian, pancreas, lung, etc. (5). The functions of FAP are mostly associated with its enzymatic activity. This can help tumor cells invade surrounding tissues, penetrate blood vessel walls, and travel to distant tissues (3). Accordingly, a high FAP-α expression can predict poor survival rates, for example, in oral squamous cell carcinoma, gastric cancer, and pancreatic cancer (4). Hence, we conducted a systematic review and meta-analysis of the available data regarding the FAP-α association with cancer metastasis.

## 2 Methods

### 2.1 Literature search strategies

The present study was performed based on the Preferred Reporting Items for Systematic Reviews and Meta-analyses (PRISMA) (6). Related studies with FAP-α and metastasis published before 20 February 2024 in PubMed, Scopus, and Web of Science were systematically included. The FAP-α keywords included “fibroblast activation protein” or “seprase” or “surface-expressed protease” or “FAPalpha” or “FAP-α” or “fibroblast proliferation factor” or “fibroblast-activating factor” or “FAP protein”, and the metastasis keywords were “metastasis” or “neoplasm metastases” or “metastase” or “lymph node metastasis” or “lymph node metastases” or “metastasis, lymph node” or “lymphatic metastases” or “nervous tissue neoplasms” or “nerve tissue neoplasms” or “blood vessel invasion”. Additional relevant searches were performed through a manual search of qualified study references to find relevant studies that linked FAP expression and metastasis.

### 2.2 Inclusion and exclusion criteria

The following outcomes were considered for the inclusion criteria (1): studies investigating FAP expression in cancer (2); studies published in English (3); studies related to human samples including human participants, body tissue samples, or human cell lines; and (4) necessary data supplied to the computation of the odds ratio (OR) with a 95% confidence interval (CI). Moreover, the exclusion criteria were as follows (1): duplicate articles (2); reviews and meta-analyses; and (3) studies that investigated only expression in the animal model.

### 2.3 Publication quality assessment

We evaluated the quality of the studies by employing the Quality Assessment Tool for Observational Cohort and Cross-Sectional Studies from the National Heart, Lung, and Blood Institute (NHLBI), National Institutes of Health (NIH) (7), which is suitable for risk of bias assessment of cohort and case–control studies (8). This is a standardized and structured tool consisting of 14 criteria that include aim description (item 1), study population description (item 2), participation rate (item 3), homogeneity of study population (item 4), sample size and power (item 5), exposure measurement (item 6), adequate timeframe (item 7), varied exposure levels (item 8), clear exposure measures (item 9), repeated exposure assessment (item 10), clear outcome measures (item 11), blinding of outcome assessors (item 12), loss to follow-up (item 13), and adjustment for confounding variables (item 14). Each criterion is assigned a binary score of 0 (absence) or 1 (presence), with additional codes for CD (cannot be determined), NA (not applicable), or NR (not reported). Two authors independently evaluated the included articles. Any disagreements were also resolved through a discussion involving all authors.

### 2.4 Data extraction

During the initial screening phase, the titles and abstracts of all collected articles were thoroughly examined to identify pertinent studies. In the subsequent screening phase, the authors extracted data from the selected studies using standard data collection forms. Before a final decision, controversial topics were discussed and compared with a third author’s opinion. Information was obtained from each study in the same format. This included the name of the first author, year of publication, country of origin, tumor type, sample size, FAP-α expression level, and OR as a measure of association. In some studies where ORs were not reported, the extracted data were analyzed to estimate ORs and 95% CIs. This was done using the OR calculation spreadsheet developed by Tierney et al. (2007) (9).

### 2.5 Statistical analysis

STATA Version 17.0 (College Station, Texas, USA) was used for all statistical analyses. The researchers employed the Restricted Maximum Likelihood (REML) method to calculate the pooled OR and their respective 95% CI. The primary objective was to investigate the association between FAP-α expression and cancer metastasis. The analyses were two-tailed, and statistical significance was considered at a *P*-value less than 0.05.

The heterogeneity of the article results was examined using the Higgins *I*-squared (I^2^) statistic.

Categorizing the heterogeneity results was carried out as follows: *I* ^2^ < 25% indicated no heterogeneity, *I* ^2^ = 25%–50% indicated moderate heterogeneity, *I* ^2^ = 50%–75% indicated large heterogeneity, and *I* ^2^ > 75% indicated extreme heterogeneity. In statistical analysis, when studies exhibit no heterogeneity, the fixed-effect model is conventionally employed. However, heterogeneous results were handled using the random-effects model. In addition, heterogeneity between subgroups was evaluated by subgroup analysis. To assess potential publication bias, a funnel plot was also created. Begg’s rank correlation and Egger’s linear regression tests were employed to quantify publication bias (10, 11). If significant publication bias was detected, a trim-and-fill analysis was conducted to evaluate the potential impact of this bias (12).

## 3 Results

### 3.1 Study and patient characteristics

**Figure 1:**
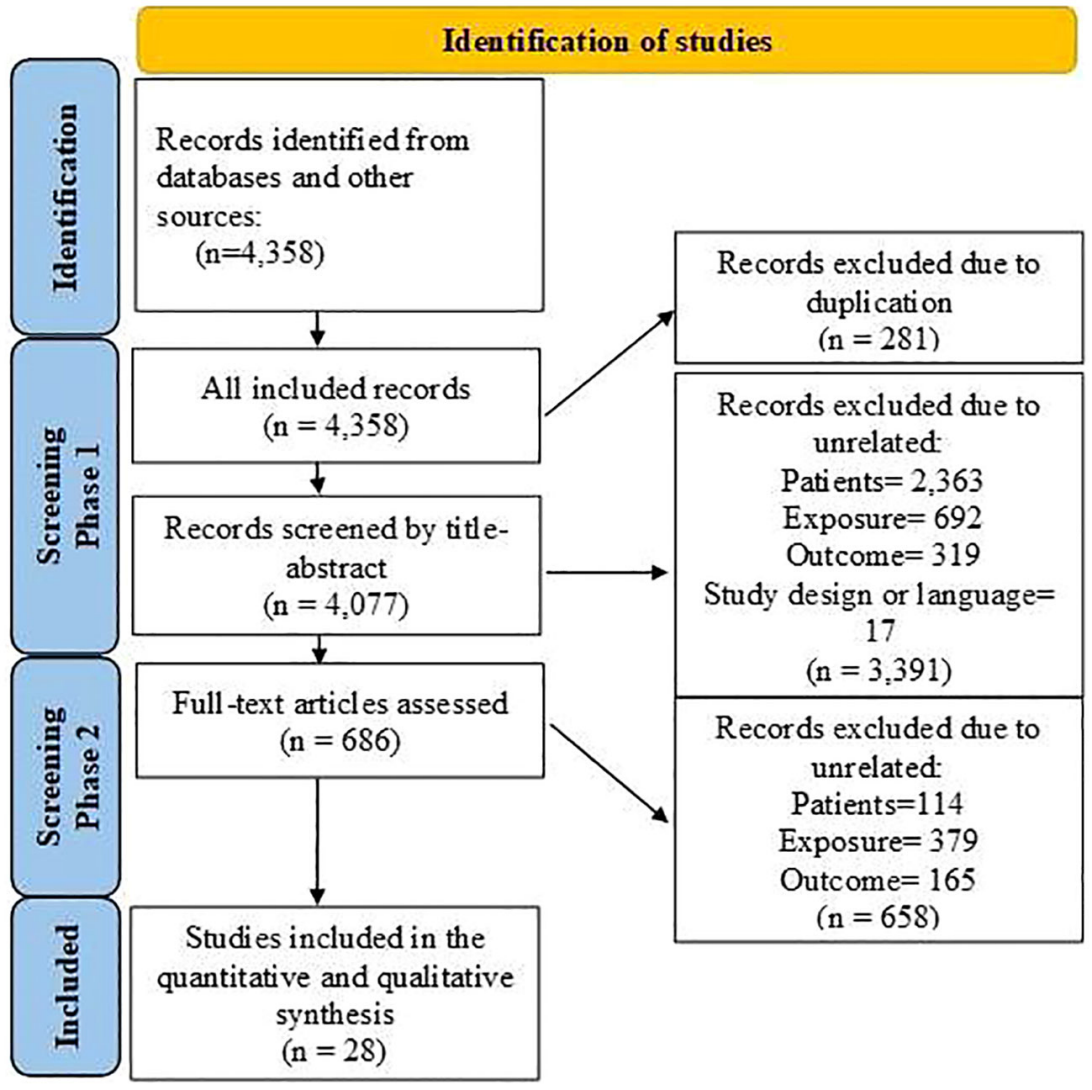
Flowchart of the selection process of studies under the guidelines outlined by the preferred reporting items for Systematic Reviews and Meta-analyses.

**Figure 1** shows that 4,358 articles were included in this systematic review, of which 281 were duplicates. After assessing the titles, abstracts, and keywords, 3,391 articles were excluded due to unrelated patient populations, exposures, or outcomes. Additionally, 686 articles that initially met the inclusion criteria were reassessed, and 28 articles (4, 13–39) were finally included in this meta-analysis. **Table 1** shows the articles published between 2007 and 2023. Among the studies conducted to determine the association between FAP-α and metastasis, 13 studies were conducted in China (4, 17, 20, 26, 27, 29, 31–36, 38), three studies in Japan (22–24), three studies in South Korea (21, 25, 30), two studies in Spain (16, 19), and seven studies in seven countries such as Sweden (15), Switzerland (18), France (14), Egypt (13), USA (37), Germany (39), and Belgium (28). The majority of the included studies were designed as cohorts, and the most common methods used for FAP-α detection were immunohistochemistry and Western blotting. The median sample size of the included studies was 113 individuals (ranging from 42 to 440). Additionally, information about the patients (cancer type), the cutoff value for FAP-α, sample size, gender proportion, mean age, and proportion of individuals with a high FAP-α level are presented in **Table 1**.

**Table 1:** Characteristics of the articles included in the study.

Study	Country	Sample size	Study design	Sex (male patients)	Mean age	Cancer type	FAP detection method	FAP cutoff	High-level FAP
Byrling et al. (2020) (15)	Sweden	122	Cohort	39	67	Distal cholangiocarcinoma	IHC	The percentage of positive cells was scored on a scale of 0–4 (0%–10%, 11%–25%, 26%–50%, 51%–75%, >76%) and the intensity of staining was scored as 0 (negative), 1 (low), 2 (moderate), and 3 (strong)	40
Chen et al. (2018) (17)	China	92	Cohort	86	NR	Lung squamous cell carcinoma	IHC	The percentage of positive cells was scored: grade 0, absent or <1% staining in the stroma: grade 1, 1%–10% positive staining; grade 2, 11%–50% positivity; grade 3, >50% positive staining. High expression was defined as a grade >2 (FAP-α positivity > 50%)	58
Coto-Lierena et al. (2020) (18)	Switzerland	59	Cohort	42 (58.69)	NR	Colorectal cancer	IHC	Tumor samples were classified into FAP-high and FAP-low groups based on the threshold of the mean + 3 standard deviations of normal tissues	58
Errarte et al. (2016) (19)	Spain	110	Cohort	45 (76%)	NR	Renal cancer	IHC and Western blot	NR	38
Gao et al. (2017) (20)	China	116	Cohort	78	57	Gastric cancer	Western blotting and IHC	The percentage of positive cells was presented by scores: no FAP and HGF protein expression: 0 points; <10%, 1 to 2 points; 10%–50%, 2 to 3 points; >50%, >3 points; substantially colorless, 0 points; light color, 1 point; dark color, 2 points. In terms of the final scores, 0 to 1 point stood for negative (–), 2–4 points for weak positive (+), 5–7 points for positive (++), 8 to 9 points for strongly positive (+++)	68
Ha et al. (2014) (21)	Korea	116	Cohort	112	NR	Esophageal squamous cell carcinoma	IHC	CAFs were divided into two groups according to their morphology on HE slides, as below (1): mature when fibroblasts show thin, wavy, and small spindle cell morphology as normal fibroblasts (2); when fibroblasts are immature, they show large, plump spindle-shaped cells with prominent nucleoli	64
Henry et al. (2007) (37)	USA	138	Cohort	67		Colon cancer	IHC	Grade 0 was defined as the complete absence or weak FAP immunostaining in <1% of the tumor stroma; grade 1+ was focal positivity in 1% to 10% of stromal cells; grade 2+ was positive FAP immunostaining in 11% to 50% of stromal cells; and grade 3+ was positive FAP immunostaining in >50% of stromal cells	101
Higashino et al. (2019) (22)	Japan	127	Cohort	NR	NR	Esophageal squamous cell carcinoma	IHC and cytokine array	FAP-positive stromal cells coexist with CD163- or CD204-positive macrophages	31
Ma et al. (2017) (26)	China	122	Case–control	68	57	Colorectal cancer	Western blotting	Based on the ratio of positive cells, scored expressions were negative (1%–10% positive cells) (–), positive (11%–50%) (+), and strongly positive (> 51%) (++)	91
Son et al. (2019) (30)	Korea	147	Cohort	88	NR	Colorectal cancer	IHC	IHC grades of FAPa in fibroblasts were measured using intensity and percentage of staining as follows: grade 1, weak staining in <50% or moderate staining in <20% of stromal cells; grade 2, weak staining in ≥50%, moderate staining in 20% to 49%, or strong staining in <20%; and grade 3, moderate staining in ≥50% or strong staining in ≥20%. IHC grades 1 to 2 were considered negative and grade 3 was considered positive	84
Song et al. (2016) (31)	China	102	Cohort	NR	NR	Ovarian cancer	IHC	The number of positive cells in no less than 3 × 100 cells was recorded. The dyeing positive rate was included for statistical analysis: the positive rate equal to or less than 95% was treated as a low expression group; otherwise, it was included in the high expression group.	61
Wen et al. (2019) (34)	China	56	Cohort	31	NR	Pancreatic cancer	IHC	The FAPα expression evaluation criteria were as follows: dyeing area ≤10% was scored as 0 points; 11% ≤25% as 1 point; >26% ≤50% as 2 points; >51% as 3 points. A negative staining intensity was scored as 0 points, weak staining as 1 point, intermediate staining as 2 points, and strong staining as 3 points. The classification of slice staining was divided according to the sum of the stained area and staining intensity score: ≤3 indicated low expression of FAP-α (FAPα negative, FAPα−); >3 indicated high expression of FAP-α (FAP-α positive, FAP-α+)	33
Yuan D. (2013) (38)	China	160	Cohort	72	NR	Osteosarcoma, corresponding non-cancerous bone tissue	IHC and Western blot	The percentage scoring of immunoreactive tumor cells was as follows: 0 (0%), 1 (1%–10%), 2 (11%–50%), and 3 (>50%). The staining intensity was visually scored and stratified as follows: 0 (negative), 1 (weak), 2 (moderate), and 3 (strong)	88
Zhang et al. (2015) (35)	China	128	Cohort	NR	NR	Ovarian carcinoma	Western blot	The ratio of the intensities of the DPPIV, FAP-α+, and GAPDH bands was recorded and divided into the following three grades: low, +; moderate, ++; and high, +++	110
Zou et al. (2018) (36)	China	138	Cohort	116	NR	Hepatocellular carcinoma	IHC, Western blot, and RT-PCR	The cutoff points were made to determine the low and high expressions of HIF-1a and FAP. Statistical significance was assessed as the cutoff score derived from the 138 cases by a standard log-rank method	74
Kashima et al. (2019) (23)	Japan	94	Cohort	79	NR	Esophageal squamous cell carcinoma	IHC and Western blot	The overall percentage of stromal FAP staining was assessed as a proportion score (0, no staining; 1, <10% staining; 2, <30%; 3, <60%; and 4, ≥60%), and the staining intensity was given an intensity score (0, none; 1, weak; 2, intermediate; and 3, strong)	50
Ambrosetti et al. (2022) (14)	France	440	Cohort	NR	NR	Renal carcinoma	IHC	NR	112
Wang et al. (2013) (33)	China	60	Case–control	36	51.5	Gastric cancer	IHC and Western blot	The degree of FAP staining in gastric cancer stroma was classified into three groups: +++, strong staining in N50% of stroma fibroblasts; ++, moderate staining in N50% of stroma fibroblasts; and +, faint or weak staining in N50% of stroma fibroblasts	24
Shi et al. (2012) (29)	China	134	Cohort	92	59	Pancreatic adenocarcinoma	Western blot	A score of 0 was assigned to a stained area with ≤10% of the tumor cells, 1 for an area with > 11% to ≤25% of tumor cells, 2 for >26% to ≤50% of tumor cells, and 3 for >51% of tumor cells	32
Wang et al. (2014) (32)	China	84	Cohort	54	54.1	Oral squamous cell carcinoma	RT-PCR and Western blot	NR	35
Calvete et al. (2019) (16)	Spain	121	Cohort	118	68	Bladder carcinoma	Microarray and IHC	Cutoff points or an automated scoring system were not used. The results of the 2 scores were combined as positive when at least 1 score was positive	76
Abd El-Azeem et al. (2022) (13)	Egypt	72	Cohort	44	64	Bladder carcinoma	IHC	The percentage scoring of positive cells was as follows: 0 (0–5%), 1 (6%–25%), 2 (26%–50%), 3 (51%–75%), and 4 (>75%). The staining intensity was scored and categorized as follows: (0 = negative, 1 = weak, 2 = moderate, and 3 = strong).	27
Kawase et al. (2015) (24)	Japan	48	Cohort	28	71	Pancreatic adenocarcinoma	IHC	FAP-positive cells were identified by IHC staining	31
Li et al. (2020) (4)	China	121	Cohort	95	64	Esophageal squamous cell carcinoma	IHC	The expression of FAP-α was found predominantly in stromal cells and slightly in cancer cells in resected ESCC tissues	45
Miao et al. (2014) (27)	China	86	Cohort	NR	NR	Gastric cancer	Western blotting	Staining was scored as per the following scale: 0, no staining; 1+, minimal staining; 2+, moderate to strong staining in at least 20% of cells; and 3+, strong staining in at least 50% of cells. Cases with 0 or 1+ staining were classified as negative, and cases with 2+ or 3+ staining were classified as positive	36
Muilwijk et al. (2021) (28)	Belgium	86	Cohort	69	NR	Bladder cancer	IHC	IHC-positive stromal area/total stromal area	22
Kim et al. (2014) (25)	Korea	42	Cohort	29	56	Hepatocellular carcinoma	IHC	IHC staining results were interpreted in a staining score, from 0 to 3, as follows: 0, staining in 5% of tumor cells; 1, weak staining in <25%; 2, moderate staining in <50%; and 3, strong staining in >50% of the tumor cells. Positive staining was defined as a staining score of 2 or 3, whereas scores of 0 and 1 were regarded as negative	28
Greimelmaier (2023) (39)	Germany	67	Cohort	34	NR	Colorectal cancer	IHC	It determined the IRS for FAP staining by combining staining intensity and the percentage of positive cells. Staining intensity was scored visually as 0 (negative), 1 (weak), 2 (moderate), or 3 (strong). The percentage of positive cells was scored as follows: 0 (none), 1 (1%–10%), 2 (11%–50%), 3 (51%–80%), and 4 (81%–100%). These scores were multiplied to calculate the IRS, which ranges from 0 to a maximum of 12. An IRS of 0 indicates FAP-negative, while IRS values of 1 to 4 represent the low expression group, and values of 5 to 12 indicate the high expression group of FAP	41

IHC, immunohistochemistry; FAP, fibroblast activation protein; HGF, hepatocyte growth factor; HE, hematoxylin and eosin; α-SMA, α-smooth muscle actin; FSP, fibroblast-specific protein; DPPIV, dipeptidyl peptidase IV; ESCC, esophageal squamous cell carcinoma; IRS, immunoreactive score.

### 3.2 Quality assessment

The quality assessment of the included studies showed a mean score of 11.07, with the highest score being 12 and the lowest score being 10. Considering that the maximum score possible on the checklist was 14, the findings suggest that the overall quality of the studies was within the range of fair to acceptable quality (**Table 2**).

**Table 2:** Quality assessment of the included studies.

First author	Item number on the checklist														Total
	1	2	3	4	5	6	7	8	9	10	11	12	13	14	
Byrling et al. (2020) (15)	1	1	1	1	1	1	1	1	1	0	1	1	NR	1	12
Chen et al. (2018) (17)	1	1	1	1	1	1	1	1	1	0	1	1	NR	1	12
Coto-Lierena et al. (2020) (18)	1	1	1	1	1	1	1	1	1	0	1	0	NR	0	10
Errarte et al. (2016) (19)	1	1	1	1	1	1	1	1	1	0	1	0	NR	0	10
Gao et al. (2017) (20)	1	1	1	1	1	1	1	1	1	0	1	0	NR	0	10
Ha et al. (2014) (21)	1	1	1	1	1	1	1	1	1	0	1	1	NA	1	12
Henry et al. (2007) (37)	1	1	1	1	1	1	1	1	1	0	1	1	NR	1	12
Higashino et al. (2019) (22)	1	1	1	1	1	1	1	1	1	0	1	1	NR	1	12
Ma et al. (2017) (26)	1	1	1	1	1	1	1	1	1	0	1	0	NA	0	10
Son et al. (2019) (30)	1	1	1	1	1	1	1	1	1	0	1	0	NA	1	11
Song et al. (2016) (31)	1	1	1	1	1	1	1	1	1	0	1	0	NR	1	11
Wen et al. (2019) (34)	1	1	1	1	1	1	1	1	1	0	1	1	NR	1	12
Yuan et al. (2013) (38)	1	1	1	1	1	1	1	1	1	0	1	1	NR	1	12
Zhang et al. (2015) (35)	1	1	1	1	1	1	1	1	1	0	1	0	NR	1	11
Zou et al. (2018) (36)	1	1	1	1	1	1	1	1	1	0	1	1	NR	1	12
Kashima et al. (2019) (23)	1	1	1	1	1	1	1	1	1	0	1	1	NR	1	12
Ambrosetti et al. (2022) (14)	1	1	1	1	1	1	1	1	1	0	1	1	NR	1	12
Wang et al. (2013) (33)	1	1	1	1	1	1	1	1	1	0	1	1	NR	0	11
Shi et al. (2012) (29)	1	1	1	1	1	1	1	1	1	0	1	0	NR	0	10
Wang et al. (2014) (32)	1	1	1	1	1	1	1	1	1	0	1	1	NR	0	11
Calvete et al. (2019) (16)	1	1	1	1	1	1	1	1	1	0	1	0	NR	0	10
Abd El-Azeem et al. (2022) (13)	1	1	1	1	1	1	1	1	1	0	1	1	NA	0	11
Kawase et al. (2015) (24)	1	1	1	1	1	1	1	1	1	0	1	0	NR	0	10
Li et al. (2020) (4)	1	1	1	1	1	1	1	1	1	0	1	1	NR	1	12
Miao et al. (2014) (27)	1	1	1	1	1	1	1	1	1	0	1	0	NR	0	10
Muilwijk et al. (2021) (28)	1	1	1	1	1	1	1	1	1	0	1	0	NR	0	10
Kim et al. (2014) (25)	1	1	1	1	1	1	1	1	1	0	1	0	NR	1	11
Greimelmaier et al. (2023) (39)	1	1	1	1	1	1	1	1	1	0	1	1	NR	0	11

CD, cannot be determined; NA, not applicable; NR, not reported.

### 3.3 Blood vessel invasion

**Figure 2:**
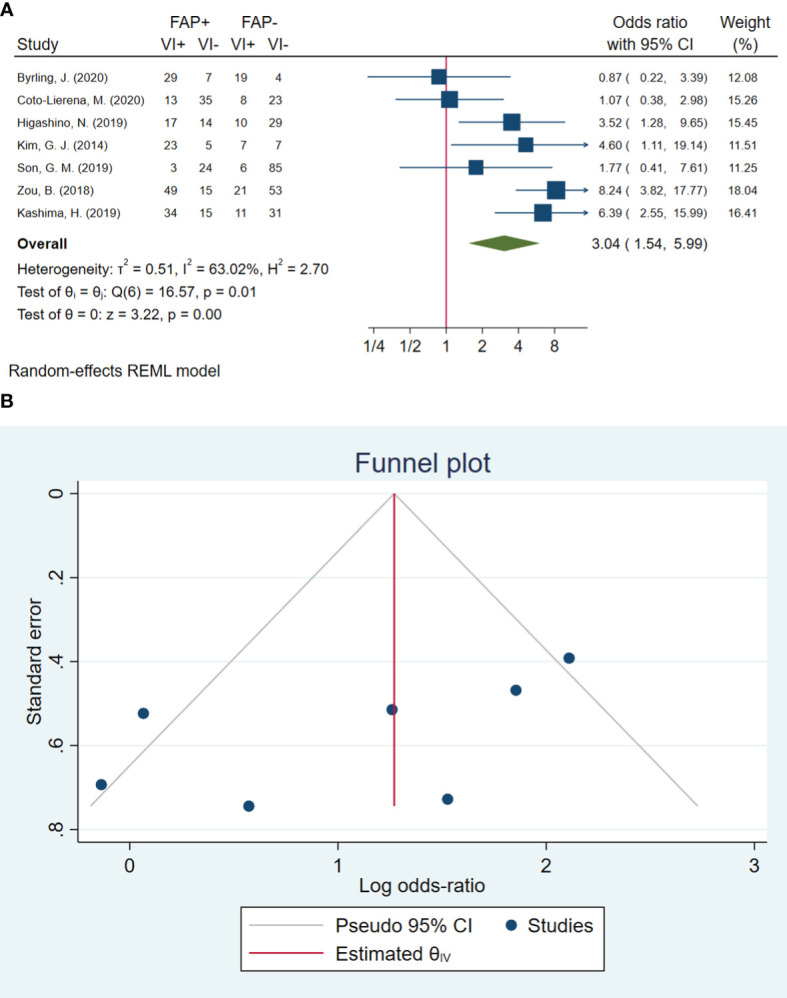
**(A)** Forest plot of studies evaluating the association between fibroblast activation protein-α (FAP-α) expression and blood vessel invasion. **(B)** Funnel plot of publication bias for comparing FAP-α expression with blood vessel invasion. VI, blood vessel invasion.

In total, seven studies involving 597 patients were conducted to evaluate blood vessel invasion. The pooled OR indicated that patients with high FAP levels had 3.04 times higher odds of blood vessel invasion than patients with low FAP levels (OR: 3.04, 95% CI: 1.54–5.99, *I* ^2^ = 63%, *P* = 0.001). The funnel plot for blood vessel invasion is shown in **Figure 2**. The Beggs (*P* = 0.230) and Egger (*P* = 0.104) tests showed no significant evidence of publication bias.

### 3.4 Lymphovascular invasion

**Figure 3:**
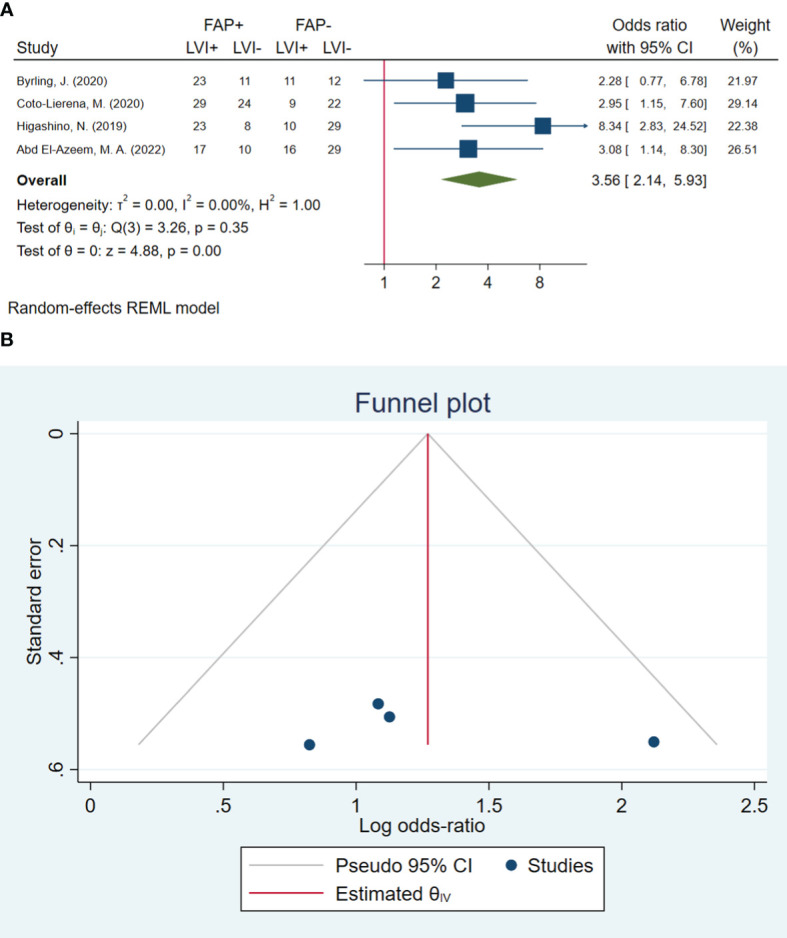
**(A)** Forest plot of studies evaluating the association between fibroblast activation protein-α (FAP-α) expression and lymphovascular invasion. **(B)** Funnel plot of publication bias for comparing FAP-α expression with lymphovascular invasion. LVI, lymphovascular invasion.

In total, four studies involving 283 patients were conducted to evaluate lymphovascular invasion. The pooled OR indicated that patients with high FAP levels had 3.56 times higher odds of lymphovascular invasion than patients with low FAP levels (OR: 3.56, 95% CI: 2.14–5.93, *I* ^2^ = 0.00%, *P* < 0.001). The funnel plot for lymphovascular invasion is shown in **Figure 3**. In addition, the Beggs (*P* = 0.999) and the Egger (*P* = 0.606) tests showed no significant evidence of publication bias.

### 3.5 Lymph node metastasis

**Figure 4:**
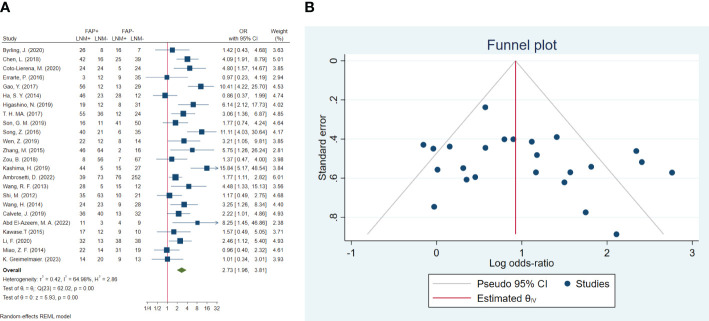
**(A)** Forest plot of studies evaluating the association between fibroblast activation protein-α (FAP-α) expression and risk of lymph node metastasis. **(B)** Funnel plot of publication bias for comparing FAP-α expression with lymph node metastasis. LNM, lymph node metastasis.

In total, 24 studies involving 2,536 patients were conducted to evaluate lymph node metastasis. The pooled OR indicated that patients with high FAP levels had 2.73 times higher odds of lymph node metastasis than patients with low FAP levels (OR: 2.73, 95% CI: 1.96–3.81, *I* ^2^ = 65%, *P* < 0.001). The funnel plot for lymphovascular invasion is shown in **Figure 4**. In addition, the Beggs (*P* = 0.309) and the Egger (*P* = 0.249) tests showed no significant evidence of publication bias.

### 3.6 Distant metastasis

**Figure 5:**
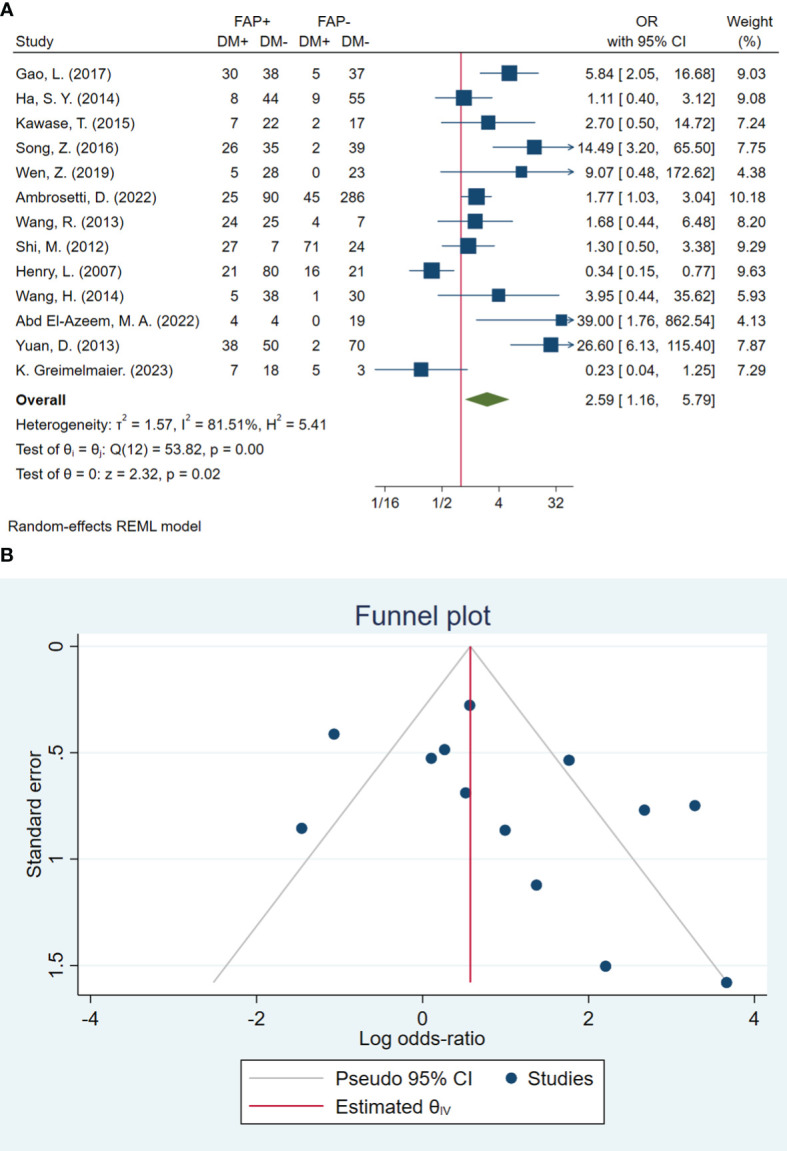
**(A)** Forest plot of studies evaluating the association between fibroblast activation protein-α (FAP-α) expression and distant metastasis **(B)**. Funnel plot of publication bias for comparing FAP-α expression and distant metastasis. DM, distant metastasis.

In total, 13 studies included 1,499 patients in assessing distant metastasis. The pooled OR showed that the odds of having distant metastasis in patients with high FAP were 2.59 times higher than in patients with low FAP (OR: 2.59; 95% CI: 1.16–5.79, *I* ^2^ = 81%, *P* < 0.001). The statistical results of the Beggs (*P* = 0.127) and Egger (*P* = 0.071) tests showed non-significant publication bias, as illustrated in **Figure 5**.

### 3.7 Neural invasion

**Figure 6:**
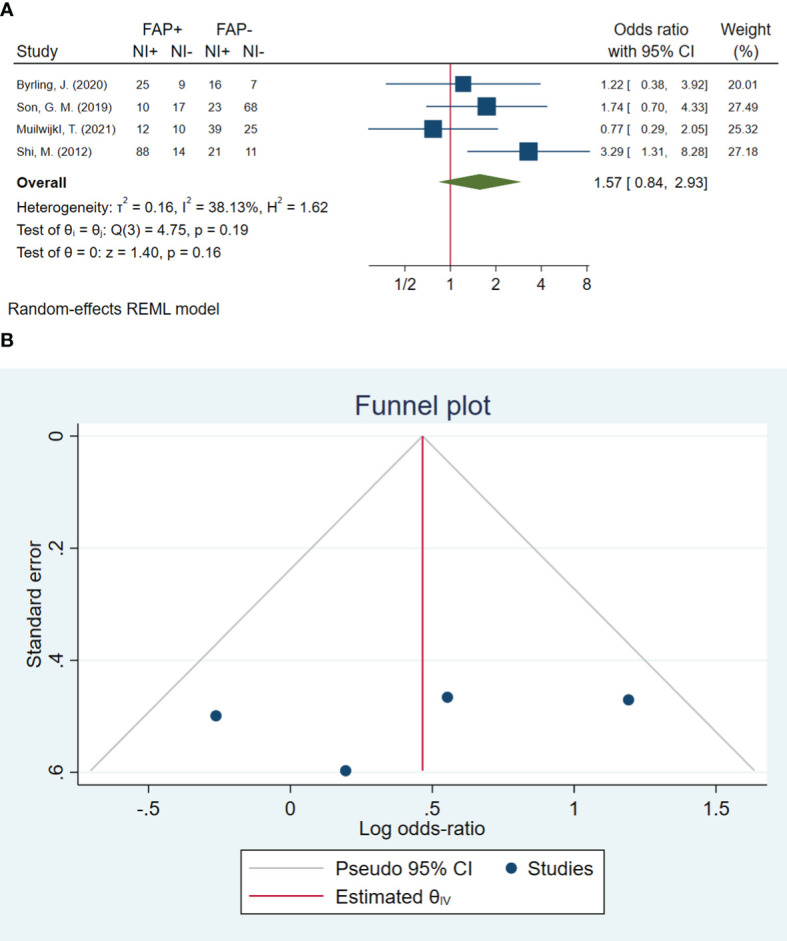
**(A)** Forest plot of studies evaluating the association between fibroblast activation protein-α (FAP-α) expression and neural invasion **(B)**. Funnel plot of publication bias for comparing FAP-α expression with neural invasion. NI, neural invasion.

In total, four studies involving 395 patients were conducted to evaluate neural invasion. The pooled OR indicated that patients with high FAP-α levels had 1.57 times higher odds of neural invasion than patients with low FAP-α levels (OR: 1.57, 95% CI: 0.84–2.93, *I* ^2^ = 38%, *P* = 0.161). The funnel plot of neural invasion is shown in **Figure 6**. The Beggs (*P* = 0.734) and Egger (*P* = 0.490) tests showed no significant evidence of publication bias.

### 3.8 Subgroup analysis

Subgroup analysis was conducted for blood vessel invasion, lymph node metastasis, and distant metastasis, which showed significant heterogeneity in the results (**Table 3**). This analysis was conducted based on total sample size, high-FAP/low-FAP ratio, FAP cutoff method, cancer type, and FAP detection method subgroups. The results of the subgroup analysis showed non-significant differences from the total sample size (*P* = 0.261), high-FAP/low-FAP ratio (*P* = 0.675), and FAP cutoff method (*P* = 0.845) subgroups of blood vessel invasion. However, we were unable to conduct the subgroup analysis of cancer type and FAP detection method with blood vessel invasion since all of the studies were carried out on gastrointestinal (GI) cancer patients and determined by the immunohistochemistry method (**Table 3**).

**Table 3:** Subgroup analysis of outcomes with heterogeneity, including blood vessel invasion, lymph node metastasis, and distant metastasis.

Subgroups	No. of studies	OR (95% of CI)	Heterogeneity *I* ^2^ (%)	*P*-value heterogeneity	*P*-value heterogeneity between subgroups
Blood vessel invasion					
Sample size					0.261
Under 100 patients	4	2.28 (0.89–5.82)	67.89	0.024	
Over 100 patients	3	4.80 (1.95–11.80)	42.93	0.179	
High/low FAP ratio					0.675
<1	2	3.85 (1.69–8.77)	0.00	0.764	
≥1	4	2.85 (0.91–8.93)	81.32	0.001	
FAP expression cutoff					0.845
By percentage of FAP	4	2.81 (1.10–7.13)	53.86	0.087	
Other methods	3	3.26 (1.01–10.53)	79.04	0.007	
Cancer type					–
GI cancers	7	3.042 (1.54–5.99)	63.02	0.011	
Detection method					–
IHC method	7	3.042 (1.54–5.99)	63.02	0.011	
Western blot	0	–	–	–	
Lymph node metastasis					
Sample size					0.831
Under 100 patients	12	2.85 (1.69–4.79)	59.79	0.004	
Over 100 patients	12	2.64 (1.69–4.13)	70.66	<0.001	
High/low FAP ratio					0.772
<1	9	2.54 (1.66–3.90)	53.22	0.034	
≥1	15	2.80 (1.73–4.52)	68.89	<0.001	
FAP expression cutoff					0.959
By percentage of FAP	10	2.87 (1.71–4.81)	60.42	0.009	
Other methods	6	2.54 (1.30–4.95)	60.47	0.028	
Not reported	8	2.70 (1.42–5.15)	76.75	<0.001	
Cancer type					0.007
Urinary tract cancer	4	1.93 (1.32–2.81)	0.00	0.286	
GI cancers	17	2.52 (1.68–3.77)	65.03	<0.001	
Ovarian cancer	2	9.07 (3.90–21.07)	0.00	0.479	
Lung cancer	1	4.09 (1.91–8.79)	–	–	
Detection method					0.351
IHC method	19	2.90 (1.98– 4.33)	67.61	<0.001	
Western blot	5	2.08 (1.13– 3.81)	50.03	0.089	
Distant metastasis					
Sample size					0.900
Under 100 patients	6	2.4 (0.69–8.56)	57.78	0.045	
Over 100 patients	7	2.70 (0.89–8.17)	89.40	<0.001	
High/low FAP ratio					0.563
<1	6	1.70 (0.95–3.02)	0.00	0.245	
≥1	6	2.77 (0.58–13.01)	88.63	<0.001	
FAP expression cutoff					0.735
By percentage of FAP	5	1.89 (0.46–7.85)	70.36	0.033	
Other methods	3	2.20 (0.25–19.73)	88.04	<0.001	
Not reported	5	3.76 (1.27–11.14)	79.79	0.002	
Cancer type					0.001
Urinary tract cancer	2	5.61 (0.30–105.65)	73.15	0.054	
GI cancers	9	1.42 (0.66–3.06)	66.98	0.001	
Ovarian cancer	1	14.47 (3.20–65.50)	–	–	
Osteosarcoma	1	26.60 (6.13–115.40)	–	–	
Detection method					0.760
IHC method	10	2.60 (0.96–7.03)	86.27	<0.001	
Western blot	3	2.07 (0.71– 6.05)	17.61	0.352	

OR, odds ratio; FAP, fibroblast activation protein; GI, gastrointestinal; IHC, immunohistochemistry.

Additionally, the results of the subgroup analysis showed non-significant subgroup effects of study sample size (*P* = 0.831), ratio of high-FAP/low-FAP (*P* = 0.772), FAP cutoff method (*P* = 0.959), and FAP detection method (*P* = 0.351) on lymph node metastasis. However, a significant difference was observed between cancer-type subgroups (*P* = 0.007). The cancer type significantly modified the FAP effects on lymph node metastasis. High-FAP ovarian cancer patients (OR: 9.07, 95% CI: 3.90–21.07), lung cancer patients (OR: 4.09, 95% CI: 1.91–8.79), and GI cancer patients (OR: 2.52, 95% CI: 1.68–3.77) had higher odds of lymph node metastasis compared to patients with urinary tract cancer. Furthermore, heterogeneity was detected among studies conducted on GI cancer patients (*I* ^2^ = 65.03%, *P* < 0.001) (**Table 3**).

Furthermore, the results of the subgroup analysis showed a non-significant subgroup effect, including sample size (*P* = 0.900), ratio of high-FAP/low-FAP (*P* = 0.563), FAP cutoff method (*P* = 0.735), and FAP detection method (*P* = 0.760) on distant metastasis. Similarly to lymph node metastasis, the test revealed a significant difference between cancer-type subgroups (*P* < 0.001). In other words, patients with high FAP and osteosarcoma cancer 26.60 (95% CI: 6.13–115.40), ovarian cancer 14.47 (95% CI: 3.20–65.50), and urinary tract cancer 5.61 (95% CI: 0.30–105.65) had higher odds of distant metastasis compared to patients with high FAP and GI cancer 1.42 (95% CI: 0.66–3.06). Additionally, the results showed heterogeneity among studies conducted in GI cancer patients (*I* ^2^ = 66.98%, *P* = 0.001) (**Table 3**).

## 4 Discussion

Our results revealed a significant association between FAP-α expression and cancer metastasis. FAP-α expression increases vascular invasion, lymphovascular invasion, lymph node metastasis, and distant metastasis in various cancers. In addition, our subgroup analysis of blood vessel invasion, lymph node metastasis, and distant metastasis showed substantial heterogeneity. This highlights the complex role of FAP expression in cancer progression. The sample size, the ratio of high to low FAP, and the FAP cutoff method had no significant impact on blood vessel invasion, lymph node metastasis, or distant metastasis. As a result, cancer type was a significant modifier, particularly for distant metastases and lymph node metastases. There was a significant increase in lymph node metastasis for ovarian, lung, and GI cancers. In addition, there was a significant increase in distant metastases in osteosarcoma, ovarian, and urinary tract cancers. This was coupled with the considerable heterogeneity observed in GI cancer studies. These findings show that FAP expression affects cancer metastasis significantly, depending on the type of cancer. More diverse research is needed to determine the effect of FAP on cancer metastasis in different cancer types.

Previous studies showed that FAP-α overexpression was seen not only in malignant cells but also in stromal fibroblasts (32). In this setting, the FAP-α deficiency has an essential role in tumor inhibition, contributing to tumor angiogenesis reduction and altered ECM remodeling (40). In addition, FAP-α expression through CAF activation causes cancer growth and metastasis (24). Consistent with our meta-analysis results, a positive correlation of FAP-α expression with lymphatic vessel density in squamous cell carcinoma of the lung was reported (17). In this respect, there is a direct association between high FAP-α expression, increased tumor grade, and poor survival rates (41). Unlike normal tissues, the expression and abundance of stromal FAP-α in esophageal squamous cell carcinoma (ESCC) are shown (4). It seems that FAP-α can act as a biomarker in cancer development because of the significant correlation between FAP-α expression in primary tumors and their corresponding local and distant metastases (42). In other words, it has been shown that high FAP-α intensity plays a crucial role in the prognosis of non-small lung cancer associated with negligible anticipation in multivariable analysis (43, 44). Moreover, FAP-α expression results in the lymphatic invasion of colorectal tumors (45, 46). All the same, there was a positive correlation between FAP-α expression at both locations and lymph metastases. In this respect, FAP-α was found to be expressed in CAFs that penetrated lymph nodes, which can be a sign of fibroblast activation related to cancer cell migration (47). Moreover, higher FAP-α levels correlate with higher tumor size and lymphovascular invasion. The present findings confirm the potential practicality of FAP-α as a biomarker of cancer progression. However, further studies will be necessary to understand the role of FAP-α in cross-communication between TME cells from primary and metastatic tumors. A novel group of positron emission tracers was introduced in 2018 (48, 49). They summarized the evidence gathered to date from patients and discussed its possible implications for radiotherapy planning. Since metastasis represents a major problem in cancer, the importance of such studies will benefit the design of more effective diagnostic, prognostic, and therapeutic approaches.

### 4.1 Limitations and clinical applications

Our study has several limitations that need to be considered. First, some outcomes had moderate to high heterogeneity. This may affect the pooled estimates’ reliability. Second, all studies reported the value of FAP in patients as a categorized variable, which potentially causes boundary effect bias. Furthermore, variations in measurement methods for FAP-α expression could introduce inconsistency in the results. The design of the included studies was mainly cohorts, and exposure was measured once, so it is critical to be cautious when attributing causality to these associations. Finally, the search was limited to studies published in English, which may introduce language bias. Therefore, further studies are necessary to assess the association between FAP-α expression and metastasis.

Clinical applications recommend assessing FAP expression in screening and risk assessment protocols. It seems that identifying individuals with elevated FAP-α expression levels can facilitate the development of personalized treatment strategies, which may include more intensive therapeutic approaches or increased surveillance. Additionally, a high FAP-α expression can prove to be a valuable tool as a prognostic marker, emphasizing the necessity for enhanced follow-up and continuous monitoring in individuals exhibiting this characteristic.

## 5 Conclusion

In summary, this meta-analysis indicated that cancer cells with high FAP-α overexpression have a higher risk of metastasis than those with low FAP-α expression. These findings support the potential importance of FAP-α as a biomarker for cancer metastasis prediction.

## Data availability statement

The original contributions presented in the study are included in the article/supplementary material. Further inquiries can be directed to the corresponding authors. 

## Author contributions

MJ: Data curation, Methodology, Software, Writing – original draft, Writing – review & editing. AP: Data curation, Formal analysis, Methodology, Writing – original draft. TA: Data curation, Software, Writing – original draft. GD: Data curation, Formal analysis, Writing – original draft. FA: Data curation, Formal analysis, Writing – original draft. YJ: Data curation, Formal analysis, Writing – original draft. FB-N: Data curation, Formal analysis, Software, Writing – original draft. ZK: Data curation, Funding acquisition, Writing – original draft. MR: Writing – original draft, Writing – review & editing. MS: Writing – original draft, Writing – review & editing. VK: Formal Analysis, Funding acquisition, Writing – original draft. AA: Data curation, Formal analysis, Project administration, Supervision, Validation, Writing – original draft, Writing – review & editing.
